# Neonatal pneumonia in sub-Saharan Africa

**DOI:** 10.1186/s41479-016-0003-0

**Published:** 2016-04-12

**Authors:** Robin J. Green, Jessica M. Kolberg

**Affiliations:** Department of Paediatrics and Child Health, University of Pretoria and Steve Biko Academic Hospital, Pretoria, South Africa

**Keywords:** Neonatal, Pneumonia, Africa, Developing countries, sub-Saharan Africa

## Abstract

Neonatal pneumonia is a devastating condition. Most deaths in sub-Saharan Africa can be attributed to preventable diseases, including pneumonia, diarrhoea and malaria, which together killed an estimated 2.2 million children under the age of 5 years in 2012, accounting for a third of all under-five deaths in this region. Some countries are making progress in reducing mortality through community-based health schemes; however, most countries in this region are far from achieving the World Health Organization Sustainable Development Goals for reducing childhood morbidity and mortality. The microorganisms causing neonatal pneumonia are well known. Both bacteria and viruses are commonly responsible, while fungal organisms occur in the context of nosocomial disease, and parasites occur in HIV-infected children. The common bacterial pathogens are group B streptococci (and other streptococcal species) and Gram-negative organisms, most notably *Escherichia coli* and *Klebsiella* spp. The viruses that predominate are the common respiratory pathogens, namely respiratory syncytial virus, human rhinovirus, and influenza virus. Viral disease is often nosocomial and transmitted to infected neonates in the neonatal intensive care unit or other neonatal facilities by infected parents and staff. Neonatal pneumonia often presents with non-specific respiratory distress in newborns. In the premature infant it is often indistinguishable from surfactant deficiency-associated respiratory distress syndrome. Therefore, diagnostic testing that is cheap and reliable is urgently sought in this region. All neonates with pneumonia must receive broad-spectrum antibiotic cover. This usually entails the combination of penicillin and an aminoglycoside. A lack of appropriate drugs and neonatal intensive care unit facilities are hampering progress in managing neonatal pneumonia.

## Background

Neonatal pneumonia (NP) is a common problem in sub-Saharan Africa. Unfortunately, this condition contributes to an enormous burden of ill-health, morbidity, and mortality. Poverty, combined with under-resourced health facilities dictate that the condition is poorly managed in many African countries. Failure to prevent and/or recognise respiratory distress in neonates, along with the unavailability of antibiotics and oxygen, creates a bleak picture for management of this issue. Attention to this condition is important if the World Health Organization (WHO) Millennium Developmental Goal 4, or Sustainable Development Goal 3 (to reduce childhood mortality) is ever to be achieved [1].

## Epidemiology

A United Nations Children’s Fund (UNICEF) report in 2013 suggested that the global under-five mortality rate has been reduced by 47 % since 1990 [2], yet this still means that 216 million children under 5 years of age died in the subsequent 23 years. Infectious diseases such as pneumonia, diarrhoea, malaria, and preventable neonatal causes (including NP), still account for the most mortality. The highest mortality rates are found in the low-income countries of sub-Saharan Africa and South Asia [2]. Sub-Saharan Africa not only has the highest under-five mortality rate in the world but also has the fastest population growth. Forty-four per cent of under-five deaths in the world (now 2.9 million every year) occur during the neonatal period, with up to 50 % of infants dying during their first day of life [2]. Over two-thirds of these deaths are preventable, even without access to intensive care facilities. Since NP is prevalent in developing countries, it has been estimated that 750,000-1.2 million neonatal deaths may be attributed annually to pneumonia; NP accounts for 10 % of the global childhood mortality, but is excessively skewed to developing countries [3].

Approximately half of under-five deaths worldwide occur in only five countries: India, Nigeria, the Democratic Republic of the Congo (DRC), Pakistan, and China [4]. Nigeria and the DRC fall within the region designated as sub-Saharan Africa. Most deaths in sub-Saharan Africa can be attributed to preventable diseases, including pneumonia, diarrhoea, and malaria, which together killed an estimated 2.2 million children under the age of 5 years in 2012, accounting for a third of all under-five deaths in this region [4]. Children are at greater risk of dying if they are born in rural areas, poor households, or to a mother who has not received a basic education [4]. Early and appropriate vaccination should help to improve outcomes.

The three leading causes of neonatal mortality are pre-term birth complications, pneumonia, and intrapartum-related complications [5]. Because of the unavailability of radiological services in many impoverished regions, many neonates with sepsis are labeled as having pneumonia, and the reverse is likewise true. Whilst reductions in pneumonia contributed to fewer neonatal deaths recorded in 2013 versus 2000, there has been limited progress in improving survival in the areas of congenital abnormalities, prematurity, neonatal sepsis, and other causes [5].

Between 2009 and 2011, a study of the leading causes of death in the Taabo Health and Demographic Surveillance System (HDSS) in south-central Côte d’Ivoire (a rural region of West Africa) revealed that of the 712 deaths analysed, maternal and neonatal conditions accounted for 8.3 % of deaths, primarily due to pneumonia (*n* = 19) and birth asphyxia (*n* = 16) in newborns [6]. However, even in this region of the world, in spite of challenges, interventions have led to mortality reductions. Niger has achieved far greater reductions in child mortality and gains in coverage for interventions in child survival than neighbouring countries in West Africa [7]. The mortality rate in children younger than 5 years declined significantly from 226 deaths per 1000 live births in 1998 to 128 deaths in 2009, an annual rate of decline of approximately 5 %. Using a Lives Saved Tool (LiST) it was estimated that about 59,000 lives were saved in children younger than 5 years. The authors attributed this decrease in child mortality to the introduction of insecticide-treated bednets (25 %); improvements in nutritional status (19 %); vitamin A supplementation (9 %); treatment of diarrhoea with oral rehydration salts and zinc, and care-seeking for fever, malaria, or childhood pneumonia (22 %); and vaccinations (11 %).

In South Africa, neonatal and childhood mortality is surprisingly high for a developing country with a well-established medical infrastructure. The top five leading causes of death in 1266 children under 5 years of age treated at the Pietersburg/Mankweng Hospital Complex in the north of South Africa were prematurity/low birth weight, pneumonia, diarrhoeal diseases, birth asphyxia, and severe malnutrition [8]. The ten most common conditions represented 73.9 % (*n* = 915) of mortality causes at this facility.

Under-five mortality is decreasing in many regions of sub-Saharan Africa but neonatal mortality rates have changed very little. Whilst the problem of maternal human immunodeficiency virus (HIV) infection is associated with much higher mortality in infancy and among older children, maternal HIV status has little effect on neonatal mortality [9]. Attention to HIV care in pregnant women would help improve child survival, but different interventions are needed to reduce neonatal mortality [9].

Over half of the countries in sub-Saharan Africa are now implementing community case management (CCM) for pneumonia, diarrhoea, and malaria [10]. There is also an important need for expansion of this modality into newborn care. National health management information systems should also incorporate CCM activities [10]. CCM involves up-skilling, supporting, and supplying community health workers to play an active part in managing sick children with limited access to care at health facilities in their communities.

A study in Namibia [11] suggested that measles virus infection during pregnancy allows for a higher risk of negative maternal, foetal, and neonatal outcomes, including maternal death. Of 42 measles infections in pregnant women, 25 (60 %) had one or more adverse event and 5 (12 %) women died. Maximising measles immunity among women of childbearing age may decrease the incidence of neonatal morbidity and mortality.

It must be emphasised that infant mortality is centred on neonatal mortality in sub-Saharan Africa and NP is a leading cause of preventable death. Attention to recognition and management of NP in this region of the world would contribute enormously to improved survival of children.

## Causes of neonatal pneumonia

The micro-organisms causing NP are well known. Both bacteria and viruses are commonly responsible, while fungal organisms occur in the context of nosocomial disease and parasites occur in HIV-infected children.

The common bacterial pathogens are those vertically acquired such as group B Streptococci (and other streptococcal species) and Gram-negative organisms, most notably *Escherichia coli* and *Klebsiella* spp. [12]. The viruses that predominate are the common respiratory pathogens, namely respiratory syncytial virus (RSV), human rhinovirus, and influenza virus [12]. Viral disease is often nosocomial and transmitted to neonates in the neonatal intensive care unit (NICU) or other neonatal facilities by infected parents and staff.

Nosocomial and ventilator-associated infections have become a matter of major concern and an important cause of morbidity and mortality in NICUs. In the Mansoura University Children’s Hospital NICU in Egypt, nosocomial infection is a significant problem [13]. Gram-negative bacteria, especially *Klebsiella* spp*.*, were the predominant causes of neonatal nosocomial infection, and this organism is a major problem in this context in other developing countries. In this Egyptian study, *Klebsiella* was found to equate to an incidence rate of 21.4 % or 13.8 infections per 1000 bed-days.

Chlamydial sepsis is recognised as a minor cause of NP in most regions of the world. However, the prevalence of chlamydia-associated infection among newborns with late NP at Kenyatta National Hospital in Kenya was found to be high (51 %), eight times more prevalent than that reported worldwide [14]. Chest radiographs appear to be a reliable diagnostic tool in this group. In this centre, the use of anti-chlamydial drugs in addition to regular antibiotics seems justifiable when a diagnosis of late NP is made, but this approach would not necessarily translate to other developing countries.

A significant concern with nosocomial infection is that the organisms are increasingly resistant to common antibiotics. In one African centre, 70 % of the Gram-positive isolates were resistant to penicillin and 90 % of Gram-negative isolates were resistant to gentamycin and ampicillin [15].

*Klebsiella pneumoniae* is the commonest organism responsible for neonatal sepsis in Port Harcourt, Nigeria, where there is an overall decline in the antibiotic susceptibility to the commonly isolated bacterial organisms [16].

There is one important South African study [17] reporting tuberculosis (TB) causing NP. From an investigation of 77 neonates, 11 with culture-confirmed perinatal TB were identified. Six of these infants were born to mothers who had HIV and TB co-infection and these infants could be classified as having congenital TB. The main clinical findings were progressive pneumonia, pyrexia, growth retardation, hepatomegaly, splenomegaly, and meningitis. Seven of their mothers had evidence of current or past TB, or had a TB contact. One neonate and two mothers died within the first 3 months after birth.

RSV is one of the commonest aetiological agents associated with acute bronchiolitis, accounting for approximately a quarter to a third of cases in sub-Sarahan Africa [18]. In the neonatal period, RSV is associated with significant morbidity and causes both pneumonia and apnoea. The incidence of RSV-associated severe acute lower respiratory tract infection in children from developing and developed countries (5.5/1000 vs 5.6/1000, respectively) is equal but the case fatality rate (CFR) is higher (2.1 % vs 0.3-0.7 %, respectively) [19]. The CFR for individual risk factors for RSV-associated disease among children with chronic lung disease is 3.5-23.0 %, congenital heart defects 2-37 %, nosocomial infection 0-12.2 %, intensive care unit admission 1.1-8.8 %, and prematurity 0.6-1.0 % [20]. The risk of mortality with RSV disease increases 3-fold in haemodynamically significant (complex disease with pulmonary hypertension) congenital heart defects [21]. HIV is associated with a 2- to 3-fold greater risk of RSV disease and also with higher case fatality proportions (12 % vs 2 % in non-HIV-infected children) [22]. Whilst maternal HIV infection is not commonly associated with early neonatal infection, a rapidly progressive form of disease has been noted in some infants [3]. This may be caused, however, by associated infections such as syphilis.

## Presentation of neonatal pneumonia

**Fig. 1:**
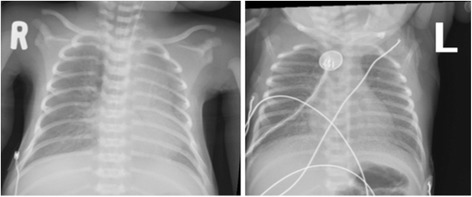
Chest radiograph of a neonate with neonatal pneumonia

NP often presents with non-specific respiratory distress in newborns. In the premature infant it is often indistinguishable from surfactant deficient respiratory distress syndrome (Fig. 1). In the full-term neonate NP may be indistinguishable from transient tachypnoea of the newborn, and meconium aspiration syndrome [23]. Fever is uncommon, whilst hypothermia is more common in the premature neonate. Pneumonia is often a component of generalised sepsis in neonates. Apnoea is a frequent clinical pattern, especially with RSV disease. A group in India have tried to define NP in a clinical setting but constellation of cough and fast breathing may not perform as well as in the older infant [3].

## Diagnosis of neonatal pneumonia

Whilst chest radiographs are valuable tools in older children and adults, in diagnosing pneumonia in the neonate, NP is frequently indistinguishable from respiratory distress syndrome. For that reason, measurement of C-reactive protein (CRP) has become a diagnostic standard. Nevertheless, from the earliest studies, CRP demonstrated no value in the early diagnosis of neonatal infection [24]. Its main role lies rather in the exclusion or confirmation of infection 24 h after the first clinical suspicion [24]. A small African study [25] suggested that absolute total neutrophil count was more reliable than CRP or procalcitonin in differentiating neonates with pneumonia. In a further study [26], mini-erythrocyte sedimentation rate was suggested to be a valuable test in the screening of the African neonate for pneumonia and sepsis. It is likely that African countries may not require sophisticated and expensive testing modalities for pneumonia. Culture of tracheal aspirates may have some value where such facilities exist but the strongest evidence for this tool lies in identification of Chlamydial disease [27].

## Prevention and management of neonatal pneumonia

Prevention of NP would be greatly assisted by simple measures such as treatment of maternal infections, careful obstetric care and general infection control measures in neonatal facilities. In a review of prevention strategies for neonatal infections, the authors suggest that there is good evidence to support the recommendations of the Centers for Disease Control and Prevention with respect to management of neonatal infections. These recommendations include maternal screening for group B streptococcus infection, intrapartum antibiotic prophylaxis, and appropriate follow-up of high-risk newborns [28]. Maternal vaccination with influenza, pertussis and potential RSV vaccines are under investigation. In the setting of HIV infection, it seems prudent to recommend better antiretroviral treatment of infected mothers and possible use of prophylactic trimethoprim-cotrimoxazole [29]. Antibiotics in this context are cheap and should be generally available in Africa. All neonates with pneumonia must receive broad-spectrum antibiotic cover which usually entails the combination of a penicillin and an aminoglycoside [30]. If *Listeria monocytogenes* sepsis occurs, the use of ampicillin is substituted for penicillin [30]. For hypoxic neonates supplemental oxygen, nasal continuous positive airflow pressure or even mechanical ventilation may be required [31]. The current WHO recommendation for treatment of severe infection in young infants is hospitalisation and parenteral antibiotic therapy; ampicillin (50 mg/kg 6 hourly) in the first week of life and an aminoglycoside (such as gentamicin 7.5 mg/kg daily) [30]. However, this recommendation is seldom practiced in sub-Saharan Africa. Hospital care is generally not available outside large cities in low- and middle-income countries and, even when available, is not accessible or affordable for many families. Previous research in Bangladesh and India [32] demonstrated that treatment outside hospitals may be possible. A program providing home visits by community health workers to diagnose and treat serious illness in young infants has demonstrated that this system functions well. However, the neonate is not usually amenable to such therapy. A lack of appropriate drugs and NICU facilities are hampering progress in managing NP in sub-Saharan Africa.

## Consequences of neonatal pneumonia

Survivors of neonatal infections are at risk of neurodevelopmental impairment. In South Asia, sub-Saharan Africa, and Latin America in late preterm neonates (more than 32-weeks gestation), through systematic reviews and meta-analyses, it was estimated that there were 1.7 million cases of neonatal sepsis, and 510,000 cases of pneumonia [33]. Moderate-to-severe neurodevelopmental impairment occurred in approximately 23 % of survivors of neonatal meningitis. Data are lacking for impairment after neonatal sepsis and pneumonia. Improved recognition and treatment of neonatal infections will reduce neonatal mortality and improve long-term outcomes. Hypothermia, pneumonia, younger gestational age, low 1 min Apgar score, and small size for gestational age are predictors of mortality and longer length of stay in the NICU [34].

Pneumonia is a common cause of hypoxia and where medical care is insufficient and resources scarce, mortality may be influenced by the unavailability of oxygen in many countries [35].

## Conclusion

NP is both common and serious in sub-Saharan Africa. It is a major contributor to under-five mortality and is currently a significant hindrance to Africa achieving the Sustainable Development Goal 3 in the near future. Basic socioeconomic factors, prevalence of HIV-infection, infectious diseases, lack of basic healthcare facilities (including vaccines, laboratory diagnostic testing and antibiotics) make a lasting solution to NP in this region of the world unlikely. Many countries in Africa have documented a turnaround in infant mortality based on simple measures and care and local government support.

## References

[1] World Health Organization MDG 4: reduce child mortality - World Health Organization. [Cited 2015 Dec 09]. Available from: www.who.int/topics/millennium_development_goals/child_mortality/en. Accessed 5 Jan 2016.

[2] Wardlaw T, You D, Newby H, Anthony D, Chopra M (2013). Child survival: a message of hope but a call for renewed commitment in UNICEF report. Reprod Health.

[3] Duke T (2005). Neonatal pneumonia in developing countries. Arch Dis Child Fetal Neonatal Ed.

[4] Guerrera G (2015). Neonatal and pediatric healthcare worldwide: A report from UNICEF. Clin Chim Acta.

[5] Liu L, Oza S, Hogan D, Perin J, Rudan I, Lawn JE (2015). Global, regional, and national causes of child mortality in 2000-13, with projections to inform post-2015 priorities: an updated systematic analysis. Lancet.

[6] Koné S, Fürst T, Jaeger FN, Esso EL, Baïkoro N, Kouadio KA (2015). Causes of death in the Taabo health and demographic surveillance system, Côte d’Ivoire, from 2009 to 2011. Glob Health Action.

[7] Amouzou A, Habi O, Bensaïd K, Niger Countdown Case Study Working Group (2012). Reduction in child mortality in Niger: a Countdown to 2015 country case study. Lancet.

[8] Ntuli ST, Malangu N, Alberts M (2013). Causes of deaths in children under-five years old at a tertiary hospital in Limpopo province of South Africa. Glob J Health Sci.

[9] Chihana ML, Price A, Floyd S, Mboma S, Mvula H, Branson K (2015). Maternal HIV status associated with under-five mortality in rural Northern Malawi: a prospective cohort study. J Acquir Immune Defic Syndr.

[10] Rasanathan K, Muñiz M, Bakshi S, Kumar M, Solano A, Kariuki W (2014). Community case management of childhood illness in sub-Saharan Africa - findings from a cross-sectional survey on policy and implementation. J Glob Health.

[11] Ogbuanu IU, Zeko S, Chu SY, Muroua C, Gerber S, De Wee R (2014). Maternal, fetal, and neonatal outcomes associated with measles during pregnancy: Namibia, 2009-2010. Clin Infect Dis.

[12] Muhe L, Tilahun M, Lulseged S, Kebede S, Enaro D, Ringertz S (1999). Etiology of pneumonia, sepsis and meningitis in infants younger than three months of age in Ethiopia. Pediatr Infect Dis J.

[13] Abdel-Wahab F, Ghoneim M, Khashaba M, El-Gilany AH, Abdel-Hady D (2013). Nosocomial infection surveillance in an Egyptian neonatal intensive care unit. J Hosp Infect.

[14] Were FN, Govedi AF, Revathi G, Wambani JS (2002). Chlamydia as a cause of late neonatal pneumonia at Kenyatta National Hospital, Nairobi. East Afr Med J.

[15] Shah AJ, Mulla SA, Revdiwala SB (2012). Neonatal sepsis: high antibiotic resistance of the bacterial pathogens in a neonatal intensive care unit of a tertiary care hospital. J Clin Neonatol.

[16] West BA, Peterside O (2012). Sensitivity pattern among bacterial isolates in neonatal septicaemia in port Harcourt. Ann Clin Microbiol Antimicrob.

[17] Adhikari M, Pillay T, Pillay DG (1997). Tuberculosis in the newborn: an emerging disease. Pediatr Infect Dis J.

[18] Pretorius MA, Madhi SA, Cohen C, Naidoo D, Groome M, Moyes J (2012). Respiratory viral coinfections identified by a 10-plex real-time reverse-transcription polymerase chain reaction assay in patients hospitalized with severe acute respiratory illness--South Africa, 2009-2010. J Infect Dis.

[19] Nair H, Nokes DJ, Gessner BD, Dherani M, Madhi SA, Singleton RJ (2010). Global burden of acute lower respiratory infections due to respiratory syncytial virus in young children: a systematic review and meta-analysis. Lancet.

[20] Welliver RC, Checchia PA, Bauman JH, Fernandes AW, Mahadevia PJ, Hall CB (2010). Fatality rates in published reports of RSV hospitalizations among high-risk and otherwise healthy children. Curr Med Res Opin.

[21] Altman CA, Englund JA, Demmler G, Drescher KL, Alexander MA, Watrin C (2000). Respiratory syncytial virus in patients with congenital heart disease: a contemporary look at epidemiology and success of preoperative screening. Pediatr Cardiol.

[22] Hall CB, Powell KR, Schnabel KC, Gala CL, Pincus PH (1988). Risk of secondary bacterial infection in infants hospitalized with respiratory syncytial viral infection. J Pediatr.

[23] Weintraub AS, Cadet CT, Perez R, DeLorenzo E, Holzman IR, Stroustrup A (2013). Antibiotic use in newborns with transient tachynea of the newborn.. Neonatalogy.

[24] Mathers NJ, Pohlandt F (1987). Diagnostic audit of C-reactive protein in neonatal infection. Eur J Pediatr.

[25] Engle WD, Jackson GL, Sendelbach DM, Stehel EK, Ford DM, McHugh KM (2003). Pneumonia in term neonates: laboratory studies and duration of antibiotic therapy. J Perinatol.

[26] Okolo AA, Scott-Emuakpor AB, Omene JA (1988). The diagnostic value of leukocyte indices and micro-erythrocyte sedimentation rate in neonatal infections. Trop Geogr Med.

[27] Sethi S, Sharma M, Narang A, Aggrawal PB (1999). Isolation pattern and clinical outcome of genital mycoplasma in neonates from a tertiary care neonatal unit. J Trop Pediatr.

[28] Reuter S, Moser C, Baack M (2014). Respiratory distress in the newborn. Pediatr Rev.

[29] Forna F, McConnell M, Kitabire FN, Homsy J, Brooks JT, Mermin J (2006). Systematic review of the safety of trimethoprim-sulfamethoxazole for prophylaxis in HIV-infected pregnant women: implications for resource-limited settings. AIDS Rev.

[30] World Health Organization. WHO. Pocket Book of hospital care for children. Guidelines for the management of common illnesses with limited resources. Second edition 2013. Geneva: World Health Organization. pp. 80-91.

[31] Wilkinson D, Andersen C, O’Donnell CP, De Paoli AG (2011). High flow nasal cannula for respiratory support in preterm infants. Cochrane Database Syst Rev.

[32] Esamai F, Tshefu AK, Ayede AI, Adejuyigbe EA, Wammanda RD, Baqui AH (2013). Ongoing trials of simplified antibiotic regimens for the treatment of serious infections in young infants in South Asia and sub-Saharan Africa: implications for policy. Pediatr Infect Dis J.

[33] Seale AC, Blencowe H, Zaidi A, Ganatra H, Syed S, Engmann C, Neonatal Infections Estimation Team (2013). Neonatal severe bacterial infection impairment estimates in South Asia, sub-Saharan Africa, and Latin America for 2010. Pediatr Res.

[34] Shah S, Zemichael O, Meng HD (2012). Factors associated with mortality and length of stay in hospitalised neonates in Eritrea, Africa: a cross-sectional study. BMJ Open.

[35] Belle J, Cohen H, Shindo N, Lim M, Velazquez-Berumen A, Ndihokubwayo JB (2010). Influenza preparedness in low-resource settings: a look at oxygen delivery in 12 African countries. J Infect Dev Ctries.

